# Integrated surveillance of antimicrobial resistance and antimicrobial use: Evaluation of the status in Canada (2014–2019)

**DOI:** 10.17269/s41997-021-00600-w

**Published:** 2022-01-31

**Authors:** Simon J. G. Otto, Margaret Haworth-Brockman, Misha Miazga-Rodriguez, Aleksandra Wierzbowski, Lynora M. Saxinger

**Affiliations:** 1 HEAT-AMR (Human-Environment-Animal Transdisciplinary AMR) Research Group, School of Public Health, University of Alberta, Edmonton, Alberta Canada; 2 Antimicrobial Resistance — One Health Consortium, Calgary, Alberta Canada; 3 Thematic Area Lead, Healthy Environments, Centre for Healthy Communities, School of Public Health, University of Alberta, Edmonton, Alberta Canada; 4 National Collaborating Centre for Infectious Diseases, Rady Faculty of Health Sciences, University of Manitoba, Winnipeg, Manitoba Canada; 5 Department of Community Health Sciences, University of Manitoba, Winnipeg, Manitoba Canada; 6 Faculty of Medicine and Dentistry, University of Alberta, Edmonton, Alberta Canada

**Keywords:** Antibiotic use, Antibiotic resistance, AMU, AMR, One Health, Surveillance, Usage des antimicrobiens, résistance aux antimicrobiens, UAM, RAM, Une seule santé, surveillance

## Abstract

**Objective:**

Integrated surveillance of antimicrobial resistance (AMR) and antimicrobial use (AMU) across One Health sectors is critically important for effective, evidence-based policy, stewardship, and control of AMR. Our objective was to evaluate progress towards achieving comprehensive, integrated AMR/AMU surveillance in Canada.

**Materials and methods:**

Based on an environmental scan, interviews of subject matter experts, and reports from the 2014 National Collaborating Centre for Infectious Diseases and the 2016 Canadian Council of Chief Veterinary Officers, we identified 8 core surveillance requirements and their specific components; the latter were assessed using a 2-way classification matrix, with 7 common elements ranked according to development stage.

**Results:**

Components that mapped to requirements of a comprehensive, fully integrated AMR/AMU surveillance system were mostly in the lowest stages of development (Exploration or Program Adoption). However, both the establishment of the Canadian AMR Surveillance System integrated reporting and expansion of existing components under the Canadian Nosocomial Infection Surveillance Program and the Canadian Integrated Program for AMR Surveillance are improvements. Regardless, obvious gaps in Canadian AMR/AMU surveillance prevent this from being a comprehensive and integrated One Health program.

**Conclusion:**

Action is needed in 3 crucial areas: i) development of a complete, integrated AMR/AMU surveillance program, based on current success; ii) changes in Federal/Provincial/Territorial policies to require standardized AMR/AMU reporting; and iii) more resources for AMR/AMU surveillance (dedicated persons, funding, and enabling structures and policy). There is an urgent need for prioritization by Federal/Provincial/Territorial governments to address governance, leadership, and funding to create surveillance systems that inform stewardship and policy.

## Résumé

**Objectif:**

La surveillance intégrée de la résistance aux antimicrobiens (RAM) et de l’utilisation des antimicrobiens (UAM) dans les secteurs Une seule santé est d’une importance cruciale pour une politique, une gestion et une maitrise efficaces et fondées sur des preuves de la résistance aux antimicrobiens. Notre objectif était d’évaluer les progrès vers la réalisation d’une surveillance complète et intégrée de la RAM/UAM au Canada.

**Méthodes:**

Sur la base d’une analyse de l’environnement, d’entrevues d’experts en la matière et de rapports du Centre de collaboration nationale des maladies infectieuses de 2014 et du Conseil canadien des chefs vétérinaires de 2016, nous avons identifié huit exigences de surveillance de base et leurs composantes spécifiques; ces derniers ont été évalués à l’aide d’une matrice de classification à deux voies, avec sept éléments communs classés selon le stade de développement.

**Résultats:**

Les composants correspondant aux exigences d’un système de surveillance de la RAM/UAM complet et entièrement intégré en étaient pour la plupart aux stades les plus bas de développement (Exploration ou Adoption de programme). Cependant, l’établissement du Système canadien de surveillance de la RAM et l’expansion des éléments existants dans le cadre du Programme canadien de surveillance des infections nosocomiales et du Programme intégré canadien de surveillance de la RAM constituent des améliorations. Quoi qu’il en soit, des lacunes évidentes dans la surveillance canadienne de la RAM/UAM l’empêchent d’être un programme Une seule santé complet et intégré.

**Conclusion:**

Des actions sont nécessaires dans trois domaines cruciaux : 1) le développement d’un programme de surveillance complet et intégré de la RAM/AMU, basé sur le succès actuel; 2) des changements aux politiques fédérales/provinciales/territoriales pour exiger des rapports normalisés sur la RAM/UAM; et 3) davantage de ressources pour la surveillance de la RAM/UAM (personnes dédiées, financement, structures et politiques habilitantes). Il est urgent que les gouvernements fédéral/provincial/territorial établissent des priorités pour aborder la gouvernance, le leadership et le financement afin de créer des systèmes de surveillance qui éclairent la gouvernance et les politiques.

## Introduction

According to the World Health Organization, antimicrobial resistance (AMR) threatens global health security (World Health Organization, 2015). Surveillance, one pillar of the World Health Organization’s Global Action Plan for AMR (World Health Organization, 2015), is endorsed by Canada in the pending Pan-Canadian Action Plan for AMR. Robust surveillance of resistant organisms and antimicrobial use (AMU) across One Health sectors is needed for effective, evidence-based policy, stewardship, and control measures in the complex interconnections among humans, animals, and the environment (World Health Organization, 2015). Integration of data across the human/animal/environment interface facilitates complete understanding of how AMU in one sector impacts AMR that can spread among all sectors.

Aspects of Federal/Provincial/Territorial (FPT) and regional AMU/AMR surveillance programs in Canada have been in existence for > 25 years (Grant et al., 2014). Core national programs, the Canadian Nosocomial Infection Surveillance Program (CNISP) (IPAC, 2020) and the Canadian Integrated Program for Antimicrobial Resistance Surveillance (CIPARS) (Government of Canada, 2020a), assemble data on resistant human nosocomial pathogens, zoonotic foodborne pathogens, and elements of AMU in both humans and animals. Although these programs provide valuable data, there are gaps in data comprehensiveness and quality (Casey, 2018; Grant et al., 2014). A lack of national-level program integration stems from challenges in sharing data, inconsistent data collection, analysis, and reporting, and limitations regarding support and infrastructure (Casey, 2018).

The most important omissions in surveillance include weak or incomplete human community-based AMR data and animal pathogen AMR data, and a lack of comprehensive AMU data for humans and animals (Casey, 2018; Grant et al., 2014). Extant human surveillance has focused on larger, urban hospitals, with gaps in data from rural and northern communities and hospitals. Animal AMR data are restricted to pathogens of concern for human health derived from food animals, but do not include pathogens from livestock and companion animals (Government of Canada, 2020a). Agricultural AMU data are limited to regional/national distribution weights and sentinel farm surveillance in a few food animal commodities. The complex Canadian distribution system for pharmaceuticals to be used in animals and previous exemptions regarding importation and use of antimicrobials, limit collation of more comprehensive data (Casey, 2018; Otto et al., 2016). Environmental AMR monitoring in soils and water is also missing, but is increasingly regarded as integral to characterizing trends and routes of transmission of AMR and an important component of One Health (Finley et al., 2013).

In 2014, the National Collaborating Centre for Infectious Diseases (NCCID) commissioned an analysis of Canadian AMR/AMU surveillance (Grant et al., 2014) with recommendations on policy, stewardship, and surveillance. In a 2016 report, the Canadian Council of Chief Veterinary Officers (CCVO) evaluated options to improve Canadian AMU surveillance in animals, with an emphasis on 3 kinds of data: FPT antimicrobial distribution/sales; veterinary antimicrobial purchase/sales/prescriptions; and animal owner antimicrobial purchase/administration (Otto et al., 2016). In addition, this report highlighted the lack of clear policy direction to inform enhancements to AMU surveillance in animals in Canada.

Our objective was to determine if progress had been made with regard to NCCID recommendations by: i) cataloguing and describing the scope of current FPT AMR/AMU surveillance programs in Canada; and ii) evaluating the current status of integrated AMR/AMU surveillance in Canada compared to gaps noted in the 2014 NCCID and 2016 CCVO reports.

## Materials and methods

Federal/Provincial/Territorial surveillance programs for AMR/AMU were identified by an environmental scan and investigator knowledge from May 1 to August 31, 2019. Yearly reports (web-based or PDF format), antibiograms, and interactive dashboards were all regarded as evidence of current and active surveillance. Subject matter experts on human and animal AMR/AMU in Canada were identified, based on involvement in programs/projects related to AMR/AMU surveillance with the NCCID and from Canadian professional networks and contacts with the authors. We conducted semi-structured interviews with these key informants to understand current Canadian FPT AMR/AMU surveillance programs in human and animal health sectors. Complete details on the environmental scan search strategy and key-informant interviews are included in the 2021 full report to the NCCID (hereafter referred to as the “full report”) (Otto et al., 2021). Additional developments were incorporated up to October 12, 2020.

The 2014 report from the NCCID made 10 broad recommendations to enhance integrated AMR/AMU surveillance in Canada (see Appendix 1 in the full 2021 report, available online; Otto et al., 2021). We modified these to 8 core AMR/AMU surveillance program requirements to represent current integrated AMR/AMU surveillance in Canada, using information from the 2016 CCVO report, environmental scan, and interviews. For evaluation, we created a 2-way classification matrix (Table 1). One classification adapted a World Health Organization situational analysis tool to quantify 5 stages of program development (Parathon et al., 2017); the second adapted a framework to rank the “sustainability capacity” of public health programs using 7 core program elements (Schell et al., 2013). We created definitions for each element and stage ranking (Table 1) and applied this 2-way classification to each component of the surveillance program. Rankings were assigned by iterative discussion within the project team based on our assessment of literature, reports, and interviews. Justifications are provided in the full report (see Supplementary Table A1, Appendix 4, available online; Otto et al., 2021). A complete description of the evaluation tool, definition, and ranking methods are described in our companion methods paper (Haworth-Brockman et al., 2021). All data were analyzed in Excel (Microsoft, Redmond, Washington, USA).

**Table 1:** Evaluation matrix for national, integrated antimicrobial resistance and antimicrobial use surveillance program component-elements in Canada. The common program elements for evaluation are in the left column and the rankings for stages of program development are in the first row. The definitions are summarized for each criteria-ranking combination, with full explanation available in Haworth-Brockman et al. (2021). Criteria are adapted from Parathon et al. (2017) and rankings are taken from Schell et al. (2013)

Common program elements	Stage of program development rankings				
	1-Exploration	2-Program Adoption	3-Initial Implementation	4-Full Operation	5-Sustainable Operation
Funding	None or limited	Basic	Time-limited, short-term	Long-term but time-limited	Permanent, dedicated funding
Organizational capacity	None or limited dedicated resources	Time-limited, dedicated resources	Time-limited, short-term, dedicated resources	Long-term but time-limited, dedicated resources	Permanent, dedicated resources
Partnerships	Relationships in earliest stages	Time-limited, formal, or informal connections with stakeholders	Short-term, time-limited, formal/informal relationships	Long-term but time-limited, formal, and informal connections	Long-term, formal connections in place
Program adaptability	No ability to improve, expand or respond to threats	Limited ability to respond	Limited ability to improve, expand and respond to threats	Moderate ability to improve, expand, and respond to emerging threats	Full flexibility to improve, expand and respond to emerging threats
Communications	Informal and limited	Limited processes and audiences in place	Periodic informal communications	Regular, periodic dissemination of program outcomes and activities	Strategic and timely communication to stakeholders, decision-makers, and public
Strategic planning	Developing	Ready for implementation	Implemented but no plan for review	Program direction, goals, and strategies in place; review process developing or in place	Fully operational with regular reviews
Enabling policy	None or in discussion stage	Developed but not implemented	Basic policy; data sharing and standardization limited or non-existent	Policies for time-limited data sharing and standardization	Policies allow effective and efficient data sharing and standardization

## Results

Our collation of the 2014 (Grant et al., 2014; Otto et al., 2021) and 2016 (Otto et al., 2016) recommendations for integrated AMR/AMU surveillance in Canada led to 8 core requirements:

1. Integrated AMR/AMU surveillance system;
2. Increased resources for AMR/AMU surveillance programs;
3. National AMR data warehouse;
4. National human AMR/AMU surveillance;
5. National animal AMR/AMU surveillance;
6. Collection of AMU indication data;
7. Timely and integrated national reporting of AMR/AMU data; and
8. Formal recognition of One Health policy for antimicrobial stewardship.

The 8 surveillance requirements were subdivided into 36 specific components (Tables 2, 3, 4, and 5) based on environmental scans, interviews, and knowledge of the investigators.

**Table 2:** Stage of development rankings for the components of integrated AMR/AMU surveillance Requirements 1 (integrated AMR/AMU surveillance system), 2 (increased resources for AMR/AMU surveillance programs), and 3 (national AMR data warehouse)

Recommended surveillance program component	Common program elements						
	Funding	Organization capacity	Partnerships	Program adaptability	Communication	Strategic planning	Enabling policy
1. Integrated AMR/AMU surveillance system							
1.1	E	E	II	E	E	E	PA
1.2	E	E	II	E	E	E	PA
1.3	E	E	II	II	E	PA	PA
2. Increased resources for AMR/AMU surveillance programs							
2.1	E	E	II	E	E	PA	PA
3. National AMR data warehouse							
3.1	FO	*N/A*	PA	II	*N/A*	FO	PA

AMR — Antimicrobial resistance; AMU — Antimicrobial use; *N/A* — Not applicable

E — Exploration; PA — Program Adoption; II — Initial Implementation; FO — Full Operation

1.1 Federally coordinated, cross-sectoral, integrated system of AMR/AMU surveillance

1.2 Standardized FPT surveillance definitions, metrics, and performance indicators

1.3 Support for integrated provincial and territorial initiatives

2.1 Resources/funding: multi-sector plan for comprehensive surveillance

3.1 AMR data warehouse (AMR NET; based on the EU model)

**Table 3:** Stage of development rankings for the components of integrated AMR/AMU surveillance Requirement 4 (national human AMR/AMU surveillance)

Surveillance program component	Common program elements						
	Funding	Organization capacity	Partnerships	Program adaptability	Communication	Strategic planning	Enabling policy
4. National Human AMR and AMU surveillance							
4.1	SO	SO	SO	FO	FO	FO	II
4.2	E	E	E	*N/A*	*N/A*	*N/A*	*N/A*
4.3	FO	FO	SO	FO	PA	FO	II
4.4	SO	SO	FO	FO	FO	FO	FO
4.5	*Project Only*	*Project Only*	*N/A*	*N/A*	*N/A*	*N/A*	*N/A*

AMR — Antimicrobial resistance; AMU — Antimicrobial use; *N/A* — Not applicable

E — Exploration; PA — Program Adoption; II — Initial Implementation; FO — Full Operation; SO — Sustainable Operation

4.1 AMR (Human nosocomial pathogens — Canadian Nosocomial Infection Surveillance Program (CNISP); foodborne pathogens in humans — Canadian Integrated Program for AMR Surveillance (CIPARS)

4.2 AMR for other human pathogens (e.g., not covered by CNISP/CIPARS, community-acquired pathogens)

4.3 Centralized collation of hospital AMU data (CNISP was the only AMU program evaluated)

4.4 Human antimicrobial distribution and prescribing data (IQVIA data)

4.5 Non-CNISP Point Prevalence Surveys of AMR/AMU in hospitals

**Table 4:** Stage of development rankings for the components of integrated AMR/AMU surveillance Requirement 5 (national animal AMR/AMU surveillance)

Surveillance program component	Common program elements						
	Funding	Organization capacity	Partnerships	Program adaptability	Communication	Strategic planning	Enabling policy
5. National Animal AMR and AMU Surveillance							
5.1	E	E	II	E	E	E	E
5.2	SO	SO	SO	SO	FO	SO	SO
5.3	SO	SO	SO	FO	FO	SO	SO
5.4	II	II	FO	FO	*N/A*	FO	FO
5.5	II	II	FO	FO	*N/A*	FO	FO
5.6	E	E	E	E	E	*N/A*	*N/A*
5.7	*N/A*	*N/A*	*N/A*	*N/A*	*N/A*	*N/A*	*N/A*
5.8	FO	FO	SO	PA	FO	FO	SO
5.9	SO	SO	SO	FO	FO	SO	SO
5.10	E	E	E	N/A	*N/A*	*N/A*	*N/A*
5.11	E	E	E	E	E	E	E
5.12	*Project only*	*Project only*	*N/A*	*N/A*	*N/A*	*N/A*	*N/A*
5.13	FO	FO	FO	PA	FO	PA	FO
5.14	FO	FO	FO	PA	FO	PA	FO

AMR — Antimicrobial resistance; AMU — Antimicrobial use*; N/A* — Not applicable

E — Exploration; PA — Program Adoption; II — Initial Implementation; FO — Full Operation; SO — Sustainable Operation

5.1 Collaborative national working group on animal AMR/AMU surveillance

5.2 Canadian Integrated Program for AMR Surveillance (CIPARS) — antimicrobial sales/distribution data for animals

5.3 CIPARS farm-level AMR/AMU — swine, broiler chickens, and turkeys

5.4 CIPARS farm-level AMR/AMU — feedlot cattle

5.5 Canadian Dairy Network for Antimicrobial Stewardship and Resistance — farm-level AMR/AMU data

5.6 Farm-level AMR/AMU — cow-calf

5.7 Veterinary or farm-level AMR/AMU for remaining food and companion animals (small animals, equine)

5.8 Department of Fisheries and Oceans collection of AMU data from aquaculture producers in Canada

5.9 CIPARS animal clinical, abattoir and retail AMR components

5.10 AMR Surveillance of veterinary pathogens

5.11 Reporting requirements for antimicrobial susceptibility data from veterinary labs (AMRNet)

5.12 AMR surveillance in soil and water

5.13 CIPARS crop AMU surveillance (Pest Management Regulatory Agency)

5.14 CIPARS farmed aquaculture AMU surveillance (Department of Fisheries and Oceans Canada)

**Table 5:** Stage of development rankings for the components of integrated AMR/AMU surveillance Requirements 6 (collection of AMU indication data), 7 (timely and integrated national reporting of AMR/AMU data), and 8 (formal recognition of a One Health policy for antimicrobial stewardship)

Recommended surveillance program component	Common program elements						
	Funding	Organization capacity	Partnerships	Program adaptability	Communication	Strategic planning	Enabling policy
6. Collection of Antimicrobial Use Indication Data							
6.1	SO	SO	SO	FO	FO	SO	SO
6.2	II	II	FO	FO	*N/A*	FO	FO
6.3	II	II	II	II	*N/A*	II	FO
6.4	II	II	PA	E	*N/A*	E	E
6.5	SO	SO	FO	PA	FO	E	PA
7. Timely and Integrated National Reporting of AMR/AMU data							
7.1	SO	SO	SO	II	FO	FO	FO
7.2	SO	PA	SO	FO	FO	SO	II
7.3	II	II	SO	*N/A*	*N/A*	SO	II
8. Formal Recognition of a One Health Policy for Antimicrobial Stewardship							
8.1	E	E	E	E	*N/A*	*N/A*	E
8.2	SO	SO	SO	SO	*N/A*	*N/A*	SO
8.3	SO	SO	SO	SO	*N/A*	*N/A*	SO
8.4	SO	SO	SO	SO	*N/A*	*N/A*	SO

AMR — Antimicrobial resistance; AMU — Antimicrobial use; *N/A* — Not applicable

E — Exploration; PA — Program Adoption; II — Initial Implementation; FO — Full Operation; SO — Sustainable Operation

6.1 Swine/broiler chicken/turkey on-farm programs provide indication data (Canadian Integrated Program for AMR Surveillance — CIPARS)

6.2 Beef feedlot indication data (CIPARS)

6.3 Canadian Dairy Network for Antimicrobial Stewardship and Resistance (CaDNetASR)

6.4 Veterinary Prescribing Surveillance (Canadian Veterinary Medical Association Stewardship of Antimicrobials for Veterinarians Initiative)

6.5 Human antimicrobial indication data (primarily Canadian AMR Surveillance System (CARSS) IQVIA Data: other sources under consideration)

7.1 CARSS — Human and animal AMR/AMU Report

7.2 CIPARS — Human and animal AMR/AMU report

7.3 CIPARS Interactive Display Dashboard for human and animal AMR/AMU reporting

8.1 Policy to recognize One Health as a priority for Canada

8.2 Legislated requirement for animal antimicrobial sales reporting (all manufacturers/importers/compounders)

8.3 Elimination of the “Own Use Importation” provision for medically important antimicrobials

8.4 Elimination of non-approved “active pharmaceutical ingredient” use and importation of medically important antimicrobials

We reviewed 6 national, 22 provincial, and 1 territorial AMR/AMU surveillance programs and data sources and we also interviewed 29 subject matter experts (following 33 invitations); see the full report for complete lists (Otto et al., 2021). National surveillance programs included the Canadian AMR Surveillance System (CARSS) (Government of Canada, 2020b), CNISP (IPAC, 2020), CIPARS (Government of Canada, 2020a), and 3 pathogen specific surveillance programs: Canadian Tuberculosis Reporting System (LaFreniere et al., 2019), AMR *Neisseria gonorrhoeae* Surveillance System (Government of Canada, 2018a), and the National Surveillance of Invasive Streptococcal Disease program (Government of Canada, 2019a).

The stage of development rankings for the components of surveillance Requirements 1 to 3 are shown in Table 2. The components of Requirement 1 (a national, integrated surveillance system) were largely in Exploration, with none ranked higher than Initial Implementation. Components of Requirement 2 (increased resources for surveillance programs) and Requirement 3 (national AMR data warehouse) ranked mostly in Exploration and Program Adoption, with few in Full Operation and none in Sustainable Operation. The major national development was the 2015 creation of CARSS, with a mandate to synthesize, integrate, and report data from human, food animal, and food AMR/AMU surveillance, under the auspices of the Public Health Agency of Canada (PHAC) (Government of Canada, 2020b). The annual CARSS report represents improvements in timely, integrated reporting, but it only includes a yearly data summary from several programs and, for example, does not reflect the complete data reported by CIPARS. Existing national surveillance programs (CIPARS and CNISP) have expanded to address gaps (Government of Canada, 2017a; Government of Canada, 2020a), but no new national, integrated surveillance program was created after 2014. However, only CIPARS was purpose-designed to provide integrated, One Health, national AMR/AMU surveillance (Government of Canada, 2020a).

Rankings for components of Requirement 4 (national human AMR and AMU surveillance refers to CNISP and subsequent CARSS data integration) are shown in Table 3. Rankings for components of Requirement 5 (national animal AMR and AMU surveillance refers to CIPARS and affiliated projects) are shown in Table 4. A large proportion of components for both requirements were ranked as Full or Sustainable Operation, illustrating the strength of CNISP and CIPARS. However, both have important gaps.

Surveillance of specific human pathogens is in Exploration, in addition to the nosocomial pathogens included within CNISP and the other select federal single pathogen-specific programs. In addition, there are various voluntary human point-prevalence hospital AMU survey systems from several, potentially competing public health-based, academic, and pharmaceutical industry-sponsored groups, with similar, albeit non-comparable data and varying degrees of overlap.

Expansions of CIPARS include components within current on-farm programs (swine, broilers, and turkeys) and the time-limited, project-funded Canadian Fed-cattle (beef feedlot) Antimicrobial Surveillance Program (Hannon et al., 2020) and the Canadian Dairy Network for Antimicrobial Stewardship and Resistance (Sanchez et al., 2018). Both receive program resources from and send data directly to CIPARS, with intentions to make them long term if funding is secured. The Health Canada Veterinary Antimicrobial Sales Reporting program currently requires annual reporting of data regarding distribution of antimicrobials for food animals and crops (Government of Canada, 2019b), with data on crop AMU from the Pest Management Regulatory Agency and data on aquaculture AMU from the Department of Fisheries and Oceans (Government of Canada, 2020a). However, there is no coordinated AMR surveillance for companion or food animal species not already included in CIPARS, nor for veterinary pathogens other than clinical non-typhoidal *Salmonella* in swine, beef, chickens, and turkeys, and some bovine respiratory pathogens in the Canadian Fed-cattle Antimicrobial Surveillance Program.

Rankings for components of Requirements 6, 7, and 8 are listed in Table 5. The Canadian Fed-cattle Antimicrobial Surveillance Program and Canadian Dairy Network for Antimicrobial Stewardship and Resistance will complement existing CIPARS on-farm AMU surveillance but remain time-limited projects (Component 6). All on-farm programs rely on a limited number of sentinel farms. Human AMU indication data come from the third-party IQVIA data provider. The annual integrated CARSS report and the annual comprehensive CIPARS report provide useful, integrated data to human and animal stakeholders, but are currently limited to electronic reports that require extensive collation that delay their release. There have been valuable changes in legislation and policy to address veterinary oversight and close loopholes in the importation of animal antimicrobials (Government of Canada, 2018b). However, there is still no formal One Health policy to address stewardship and surveillance, despite frequent program commitments to a One Health approach (Component 8) and a stated commitment to One Health in the pending Pan-Canadian Action Plan for AMR under the AMR Framework (Government of Canada, 2017b).

## Discussion

We present an evaluation of a national, integrated, One Health surveillance system for AMR/AMU in Canada. Using our novel evaluation tool (Haworth-Brockman et al., 2021), we identified that most areas of strength are within pre-existing surveillance programs (CIPARS and CNISP) (Government of Canada, 2020a; IPAC, 2020). However, the recommended components of a national, integrated AMR/AMU surveillance program are still largely in early stages of development. In that regard, there is a lack of a federally coordinated, cross-sectoral, integrated system for surveillance of AMR/AMU in Canada.

The national and global focus on AMR mitigation and surveillance has intensified since the 2014 NCCID (Grant et al., 2014) and 2016 CCVO reports (Otto et al., 2016). The recent Canadian Council of Academies (CCA) study predicted profound socioeconomic consequences if AMR is not addressed in Canada (Council of Canadian Academies, 2019). The Pan-Canadian AMR Framework recognized the need for integrated AMR/AMU surveillance as 1 of 4 key pillars (Government of Canada, 2017b) that will inform a federal action plan. Integrated, One Health AMR/AMU surveillance is critical to track AMR, inform policies on antimicrobial stewardship and clinical decision-making, establish benchmarks, and evaluate policies and actions regarding stewardship and mitigation of resistance.

During the last 6 years, Canada has made modest gains towards national, One Health, integrated AMR/AMU surveillance, with our surveillance “patchwork” improving. Integration of annual reporting within CARSS (Government of Canada, 2020b) brings CNISP and CIPARS data together, but it is a high-level summary that omits some data. It remains a data integration program, with most new components added to CNISP and CIPARS beneath it (Government of Canada, 2020a; IPAC, 2020). These additions provide some new data for AMR/AMU surveillance, including new sites for CNISP in various northern and non-tertiary Canadian hospitals providing data for otherwise unmonitored populations. A project under development through the National Microbiology Laboratory, AMRNet, could improve collation and reporting of community microbiology lab data (Government of Canada, 2016), but the current vision relies on voluntary collaboration. AMRNet is distinct from the new AMR Network project, supported by PHAC, to create a governance structure and coordinated Canadian response to AMR (AMR Network, 2020). With human AMR/AMU data largely collected through CNISP in hospitals (Government of Canada, 2017a), there are still large gaps for community human pathogen AMR surveillance focused on community-based bacterial isolates. Despite some improvements, further expansion of surveillance of hospital, long-term care, and northern and Indigenous communities is needed. In addition, relying on third-party sources of data for human community-based AMU limits the adaptability and long-term security of data availability; consequently, methods to use existing provincial data sources are needed.

Nationally, CIPARS is the sole program purpose-designed for integrated AMR/AMU surveillance that includes most One Health sectors (Government of Canada, 2020a). It has strong animal AMU and farm-to-fork components for foodborne pathogens from on-farm programs (swine, broilers, turkeys, and more recently, dairy and feedlot cattle), abattoir, animal clinical, retail food (currently pork, chicken, beef, and turkey), and human cases. However, its current components are not comprehensive. On-farm programs include a relatively limited number of sentinel farms within the provinces where they are active. It is likely that these farms do not necessarily truly represent all Canadian farms for that commodity, and for some commodities (e.g., swine — only grower-finisher operations), they do not include the complete production chain. There are also gaps in surveillance for AMR in organisms from retail foods, such as produce and aquaculture. There are still large gaps for animal pathogen data other than clinical *Salmonella* isolates and bovine respiratory pathogens in Canadian Fed-cattle Antimicrobial Surveillance Program. New feedlot and dairy components substantially expand livestock coverage, but their funding remains time-limited with uncertainty regarding future resources. Beyond crop and aquaculture AMU data, there are no environmental AMR components for water or soils to complete One Health transmission pathways, despite mounting evidence that the environment may be involved as a reservoir for AMR maintenance (Finley et al., 2013). Strategic surveillance to identify links among humans, animals, water, soils, and the broader environment are increasingly important to understand and reduce dissemination and transmission of AMR.

Timely communications are important for AMR/AMU surveillance in Canada. The current annual reports of collated, multi-program data require substantial investments in time and human capital that are not well resourced (Government of Canada, 2019c; Government of Canada, 2020b), resulting in substantial delays (often years) between data collection and reporting. The annual CARSS reports contain only a summary of CIPARS, CNISP, and other data, and annual manual reporting is an impediment to surveillance data being used in real-time (Government of Canada, 2020b). A proposed CIPARS interactive data platform would be an important advancement for timely visualization and customization of national reporting as a supplement to annual CIPARS reports. It is uncertain whether this technology will be expanded to other human surveillance reporting. However, for both human and animal health, rapid data synthesis and display or more frequent release are required to inform stewardship and AMR mitigation efforts.

Regarding action for integrated AMR/AMU surveillance, our review highlighted 3 critical areas for action: i) development of a complete, integrated AMR/AMU surveillance program, expanding from current success; ii) changes in FPT policies to compel autonomous but standardized Provincial/Territorial (PT) AMR/AMU reporting, potentially in a managed network model (Ferlie et al., 2013); and iii) investment in AMR/AMU surveillance resources (dedicated personnel, funding, and enabling structures and policy). Strong underlying policies addressing surveillance, antimicrobial stewardship, and AMR mitigation must direct the actions to achieve these elements, with the imminent Pan-Canadian Action Plan for AMR expected to provide guidance. Improvements in reporting and redistribution of resources within the patchwork of Canadian FPT surveillance activities will not create a truly comprehensive, integrated system without considering structure and having adequate resources. Furthermore, the design of this surveillance system must be guided by as well as inform stewardship policy.

A major weakness of the current FPT structure for animal and human AMR/AMU data is the combination of essential and voluntary reporting (Government of Canada, 2017b; Grant et al., 2014; Pan-Canadian Public Health Network, 2016). Compulsory reporting is restricted to animal antimicrobial sales/distribution and human foodborne diseases (*Salmonella* and *Campylobacter*). With most human and animal surveillance relying on voluntary relationships among PT health systems and academic collaborations, standardization is a challenge. Currently, British Columbia is the only province that collates province-level human antibiogram data (BCCDC, 2020). However, national surveillance requires consensus in FPT policies for data standardization, data-sharing agreements for national reporting, and a centralized data warehouse for collation and optimal real-time reporting. The CNISP relies on academic infection prevention and control professionals and liaisons with PT public health systems to collect selected AMR/AMU data, and currently third-party entities are contracted to provide community AMU data. Collated human pathogen AMR data beyond nosocomial organisms remain lacking, as do any data on veterinary pathogens. In the absence of a design-built national, integrated system with supporting policy, evolving current networks to compile PT data similar to the European Antimicrobial Resistance Surveillance Network (EARS-NET, 2019) and European Surveillance of Veterinary Antimicrobial Consumption (European Medicines Agency, & European Surveillance of Veterinary Antimicrobial Consumption, 2017) is feasible, but only with ongoing, centralized support to collect and collate standardized data.

Multiple, potentially competing point prevalence surveys from academic, public health, and pharmaceutical industry sponsors illustrate how uncoordinated activities may expand to fill current gaps, in this case in hospital-based surveillance data (Becton Dickinson and Company, Sinai Health System-University Health Network Antimicrobial Stewardship Program, & National Centre for Antimicrobial Stewardship, 2019; Mitchell et al., 2019; Versporten et al., 2018; World Health Organization, 2020). These initiatives encourage Canadian hospitals and health systems to participate, and can generate useful facility-based data, trends, and limited comparisons. However, their lack of comparability for Canadian data (e.g., variable inclusion of primary/secondary/tertiary/pediatric hospitals, kind of wards, countries, AMR and/or AMU data, as well as government versus pharmaceutical industry sponsorship) is an impediment to standardized national surveillance. Optimally, a single system or data standard for hospital prevalence data should be developed to inform Canadian antimicrobial stewardship efforts. For community AMR data — a major gap — the proposed AMRNet platform (Government of Canada, 2016), distinct from the AMR Network governance structure (AMR Network, 2020), could be truly additive, but design, participation, and rollout are incomplete and participation will likely be voluntary. The CIPARS farm programs are robust, but not comprehensive for livestock production systems across all provinces and territories. Animal pathogen data, with a few notable exceptions, and all companion animal data, are lacking.

Annual direct Canadian costs of AMR in hospitals in 2018 was estimated to be $1.4 billion (Council of Canadian Academies, 2019). In the absence of efforts to reduce AMR, this could reach $7 billion annually by 2050, with projected cumulative hospital costs of $120 billion and a $388 billion reduction in GDP. From 2013 to 2018, annual estimated PHAC expenditure for programs and staffing related to AMR was $8.5 million, ~1.4% of the annual budget (Health Canada, & Public Health Agency of Canada, 2019). Therefore, this public health AMR containment funding is 0.6% of the annual direct healthcare cost of AMR. However, this excludes other FPT investments from the Canadian Food Inspection Agency, Agriculture and Agri-Food Canada, research funding agencies, and PT governments. Regardless, projected costs far outweigh the current federal investment in AMR control, including surveillance. Given the projected future costs of AMR, there is ample latitude to consider joint FPT and partner investment in AMR mitigation through antimicrobial stewardship that is supported by comprehensive, integrated AMR/AMU surveillance. It was recently reported that Canadian AMR policy efforts were disjointed and inadequate and lacked announcement of new domestic funding beyond research grants (Van Katwyk et al., 2020); furthermore, a lack of substantial investment in AMR by the federal government in the last decade (Health Canada, & Public Health Agency of Canada, 2019), particularly in coordinated national, integrated AMR/AMU surveillance, supported this conclusion. Current surveillance programs, with static or decreasing budgets, have relied on time-limited project funding to expand current programs. The Canadian AMR Network project, supported by PHAC to develop a national governance structure (AMR Network, 2020), represents new support for coordinated AMR mitigation. However, it is only an initial part of a long and complex process and does not increase aspects of national, integrated AMR/AMU surveillance.

The urgent, required investment by FPT governments on COVID-19 dwarfs the program, personnel, and infrastructure investments needed for national, integrated AMR/AMU surveillance; however, these needs should not be forgotten during the current pandemic. Antimicrobial resistance has affected health as long as we have used antibiotics in human and animal health and food production and will undoubtedly continue once the pandemic is over. COVID-19 could worsen AMR concerns, due to altered patterns of AMU for both related and unrelated conditions (Nieuwlaat et al., 2021). Antimicrobial use for pneumonia, rising virtual care, and potential impacts of extensive cleaning and disinfection/sanitization, create AMR concerns that will require robust, integrated AMR/AMU surveillance. Although it will be important to continue to leverage and coordinate various research projects and programs that have arisen in focal attempts to fill surveillance gaps, these external efforts will not sustain, integrate, or expand current programs. It is unknown whether the Pan-Canadian AMR Action Plan will provide directed government resources for truly comprehensive, integrated national surveillance.

Our evaluation focused on national programs. However, some components included data from PT programs. Most national surveillance programs do not integrate PT AMR/AMU data; in that regard, PT data integration is voluntary and not standardized and there is a paucity of publicly available, public health-sourced community AMR data. Most are variably reported antibiograms from private or public microbiology laboratories (Otto et al., 2021). There is no standardization for testing methodology, case definition, data collection, or reporting, with BC being the only province to collate these data within public health reporting (BCCDC, 2020). Notable exceptions are CNISP data and the human *Salmonella* isolates provided to CIPARS for antimicrobial susceptibility testing and reporting by provincial laboratories (Government of Canada, 2020a; IPAC, 2020).

Evolution of core surveillance programs for Canadian AMR/AMU and integrated annual reporting with CARSS are clear progress towards comprehensive, integrated AMR/AMU surveillance. However, our evaluation revealed systemic limitations in timely, comprehensive, and integrated AMR/AMU surveillance in Canada. These are potential weaknesses in Canada’s development of an effective AMR response, given the foundational role of surveillance in control. There is an urgent need for leadership and governance by FPT to improve AMR/AMU surveillance and AMR mitigation by developing multi-stakeholder oversight groups, standardized data protocols, and data-sharing arrangements. The current FPT structure for healthcare regulation and program delivery, as well as surveillance and animal health regulation, are complex and variable. Visible prioritization within government to address public health FPT governance, leadership, and funding is required.
